# Real-world use of recombinant porcine sequence factor VIII in the treatment of acquired hemophilia A: EU PASS

**DOI:** 10.1177/20406207241260332

**Published:** 2024-09-02

**Authors:** Wolfgang Miesbach, Nicola Curry, Paul Knöbl, Charles Percy, Rita Santoro, Alvin H. Schmaier, Karolin Trautmann-Grill, Kayode Badejo, Jie Chen, Masoud Nouri, Pooja Oberai, Robert Klamroth

**Affiliations:** Haemophilia Centre, Medical Clinic 2, University Hospital Frankfurt, Theodor-Stern-Kai 7, 60590 Frankfurt am Main, Germany; Oxford Haemophilia and Thrombosis Centre, Oxford University, Oxford, UK; Department of Medicine 1, Division of Hematology and Hemostasis, Medical University of Vienna, Vienna, Austria; Queen Elizabeth Hospital Birmingham, University Hospital Birmingham, Birmingham, UK; Hemostasis and Thrombosis Unit, Azienda Ospedaliero Universitaria Renato Dulbecco, Catanzaro, Italy; University Hospitals Cleveland Medical Center, Case Western Reserve University, Cleveland, OH, USA; Universitätsklinikum Carl Gustav Carus der Technischen Universität Dresden, Dresden, Germany; Takeda Development Center Americas, Inc., Cambridge, MA, USA; Takeda Development Center Americas, Inc., Cambridge, MA, USA; Takeda Development Center Americas, Inc., Cambridge, MA, USA; Takeda Development Center Americas, Inc., Cambridge, MA, USA; Department for Internal Medicine, Vascular Medicine and Hemostaseology, Vivantes Klinikum Friedrichshain, Berlin, Germany; Institute of Experimental Hematology and Transfusion Medicine, University Hospital Bonn, Medical Faculty, University of Bonn, Bonn, Germany

**Keywords:** antibodies, drug resistance, factor VIII, hemophilia A, hemostasis

## Abstract

**Background:**

Recombinant porcine factor VIII (rpFVIII; susoctocog alfa) is indicated for the treatment of bleeding events (BEs) in adults with acquired hemophilia A (AHA).

**Objectives:**

To assess the safety, utilization, and effectiveness of rpFVIII in clinical practice.

**Design:**

EU post-authorization safety study (PASS) (NCT03199794) was a multicenter, noninterventional, post-authorization safety study conducted in adults with AHA.

**Methods:**

Data were collected retrospectively or prospectively for up to 180 days after the last rpFVIII dose. The primary objective was safety, as assessed by adverse events (AEs), serious AEs (SAEs), and AEs of special interest (AESIs) (e.g. immunogenicity, hypersensitivity reactions, thromboembolic events). Secondary endpoints included immunogenicity, rpFVIII hemostatic effectiveness, and rpFVIII utilization.

**Results:**

Fifty patients were enrolled; 31 completed the study. The median (range) follow-up for patients who completed or discontinued the study was 178 (26–371) days. The median (range) first dose of rpFVIII was 54.0 (11–200) U/kg. Thirty patients reported 46 SAEs; 5 SAEs were considered probably related to rpFVIII, of which 1 was lack of rpFVIII efficacy, and 4 were AESIs: drug resistance due to FVIII inhibition (one patient), antibody test positive for anti-pFVIII inhibitors (one patient), and *de novo* anti-pFVIII inhibitors (two patients). No hypersensitivity reactions or thromboembolic events were reported. Of the 50 initial BEs, 37 resolved [in a median (interquartile range) of 8.0 (4.0–16.0) days].

**Conclusion:**

Results from this real-world study support the use of rpFVIII for AHA, aligning with findings from the clinical trial of rpFVIII (NCT01178294) in the treatment of BEs in adults with AHA.

**Trial registration:**

EUPAS16055; NCT03199794.

## Introduction

Acquired hemophilia A (AHA) is a rare autoimmune disease characterized by inhibitory autoantibodies against endogenous factor VIII (FVIII).^1^ Treatment of AHA is based on controlling bleeding and eradicating inhibiting antibodies.^2^ Bypassing agents (BPAs) control bleeding by avoiding the need for FVIII to generate thrombin.^3^ These agents carry a risk of thromboembolic events that may be enhanced by patients’ comorbid conditions.^1,4^ High-dose replacement therapy with human FVIII (hFVIII) concentrates is used to increase FVIII levels when inhibitor titers are low [<10 Bethesda units (BU)/mL]. Otherwise, BPAs are used combined with immunosuppression.^5^ Rarely, hFVIII can be used for patients with inhibitors >10 BU/mL combined with plasmapheresis or immunoadsorption.^4,6^ Newer approaches are biochemical rebalancing agents such as emicizumab, a monoclonal antibody that mimics the function of activated FVIII by enhancing the interaction between FIX/FIXa and FX/FXa.^7^ Immunosuppression is necessary to eradicate the autoantibodies in AHA alongside all treatments that stop bleeding, but relapses occur in 15–33% of cases after stopping treatment.^8^

Recombinant porcine FVIII (rpFVIII; susoctocog alfa, Obizur^®^; Baxalta US Inc., a Takeda company, Lexington, MA, USA) is indicated for the treatment of bleeding events (BEs) in adults with AHA in the EU, UK, and USA.^9,10^ The rationale is that rpFVIII has sufficient sequence homology to hFVIII to be hemostatic but is different enough to be less susceptible to inactivation by circulating FVIII inhibitors.^11^ Treatment guidelines for AHA recommend using BPAs or rpFVIII as first-line therapy, with the final choice determined by anti-porcine FVIII (pFVIII) inhibitor titer, treatment cost, and availability.^12,13^

The safety and hemostatic efficacy of rpFVIII in patients with AHA has been demonstrated in a phase II/III, prospective, multicenter, open-label study (NCT01178294).^14^ In that study, a total of 86% (24/28) patients had successful treatment of the initial BE (complete cessation of bleeding). We present the results of an open-label, observational, post-authorization safety study (PASS) conducted in the EU, UK, and USA designed to assess the safety, effectiveness, and utilization of rpFVIII in real-world clinical practice.

## Methods

### Study design

This PASS (EUPAS16055; NCT03199794) was a multicenter, prospective and retrospective, uncontrolled, open-label, single-cohort, observational study. It was conducted between 14 December 2016 and 30 July 2021 at 15 sites in 8 countries (Austria, Germany, Switzerland, Netherlands, Italy, France, UK, and USA). Patients were followed for 180 days after the last administration of rpFVIII. In case of a new or rebleeding event treated with rpFVIII, the patient was followed for an additional 180 days (Supplemental Figure 1).

### Study population

Eligible patients were adults with AHA who had received or were receiving rpFVIII in routine clinical practice. Patients with a known anaphylactic reaction to rpFVIII, excipients, or hamster protein were excluded. Patients who had participated, within 30 days of enrollment, in a clinical study of a medicinal product/device or were scheduled to participate in such a study during this study were also excluded, as were those participating in the US post-marketing study, NCT02610127.

Informed consent was obtained from the patient or legal representative at the earliest opportunity within 30 days (prospective patient) or >30 days (retrospective patient) after rpFVIII administration (Supplemental Figure 1). If the patient died before consent was obtained, informed consent was obtained from the next of kin in accordance with local regulations. If such consent could not be obtained, data were anonymized to ensure data privacy, if permitted by local regulations.

### Outcomes and assessments

The primary objective was to document the safety of patients treated with rpFVIII. Secondary objectives were to monitor rpFVIII use in real-world clinical practice, including immunogenicity; hemostatic effectiveness of rpFVIII for resolution of BEs (bleeding stopped as assessed by the physician); rpFVIII treatment details; concomitant medication use; and complete remission rate (inhibitor eradication). An exploratory objective was the association of anti-pFVIII immunogenicity with a lack of therapeutic effect and/or other clinical manifestations.

Where available, the following data were collected: safety as assessed by adverse events (AEs), serious AEs (SAEs), and AEs of special interest (AESI; reported with the same priority as SAEs). AESIs that were primary endpoints were hypersensitivity reactions, thromboembolic events, and dose dispensing medication errors. Immunogenicity was a secondary endpoint AESI corresponding to the development of *de novo* anti-pFVIII inhibitors or an increase in anti-pFVIII inhibitors and/or anti-hFVIII titers from pretreatment levels. Overall bleeding control was assessed to determine whether bleeding stopped or did not stop. Also collected were AHA medical history; bleed description; comorbidities; concomitant medications; and rpFVIII utilization. The following laboratory parameters were collected for each treatment course of rpFVIII, where available: FVIII activity (FVIII:C), anti-hFVIII and anti-pFVIII antibody titers, and a complete blood count with differential. Physicians could use a certified laboratory to process anti-hFVIII and anti-pFVIII inhibitors.

SAEs and AEs were recorded as observed by physicians and categorized by Medical Dictionary for Regulatory Activities (MedDRA) preferred terms, with severity assessments based on physicians’ judgment. Data were extracted from existing patient medical records. Sites were recommended to enter prospective data into the electronic data capture system within 14 days of entering the patient chart, or retrospective data within 14 days of signing the informed consent form.

### Statistical analysis

The full analysis set included all patients who met the eligibility criteria. Available data for prospectively and retrospectively enrolled patients were analyzed together, with the baseline defined as the time of the first rpFVIII infusion for a new BE. To reduce potential selection bias, inclusion criteria were left broad and exclusion criteria were limited; investigators were instructed to invite all eligible patients to participate in the study; and the decision to administer rpFVIII was made by the physician before, and independently of, the invitation to participate in this study.

A minimum target of 50 patients was calculated to provide substantial safety information on real-life use of rpFVIII based on the rate of anti-pFVIII inhibitors in the phase II/III study (NCT01178294).^14^ Descriptive statistics were used to characterize the overall patient population, including BEs, treatment outcomes, and AEs. Incidence rates and 95% confidence intervals were estimated based on a Poisson regression model (intercept only). The association between rpFVIII immunogenicity and lack of therapeutic effect (BE not stopped) was assessed using Fisher’s exact test. Analyses were performed using SAS^®^ version 9.4 (SAS Institute, Cary, NC, USA).

## Results

### Patients

**Figure 1.**
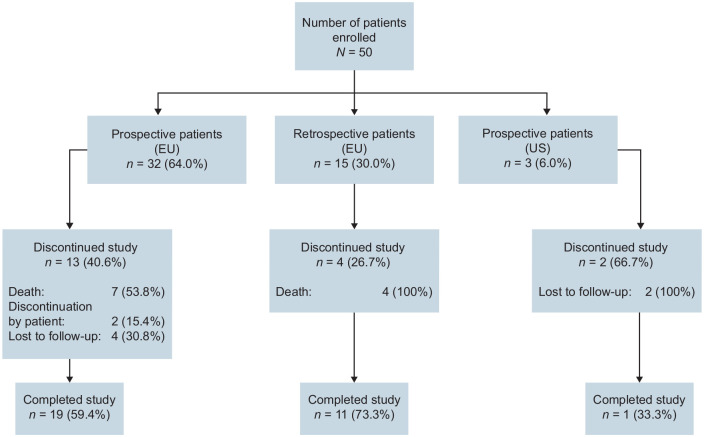
Patient disposition.

A total of 50 patients were enrolled (prospective, *n* = 35; retrospective, *n* = 15), of whom 31 (62.0%) completed the study and 19 (38.0%) discontinued. Among the 19 patients who discontinued, the primary reason for discontinuation was death (*n* = 11) (Figure 1). Causes of death were cerebral hemorrhage (*n* = 1), multiple organ failure (*n* = 1), heart disease (*n* = 2), liver failure (*n* = 1), sepsis (*n* = 1), suspected infection (*n* = 1), pneumonia (*n* = 2), and natural cause or unknown reason (*n* = 2). The median (range) follow-up for patients who had completed or discontinued the study was 178 (26–371) days. Overall, most enrolled patients were male and were ⩾65 years of age (Table 1).

**Table 1.** Baseline demographics.

Characteristic	*N* = 50
Age at initial rpFVIII treatment, mean (SD) years	75.5 (10.25)
Age category, *n* (%)	
18–64 years	6 (12.0)
⩾65 years	44 (88.0)
Sex, *n* (%)	
Male	30 (60.0)
Female	20 (40.0)
Race, *n* (%)	
Asian	1 (2.0)
Black	1 (2.0)
White	25 (50.0)
Mixed/multiple races	1 (2.0)
Not available	22 (44.0)
Ethnicity, *n* (%)	
Not Hispanic or Latino	28 (56.0)
Not available	22 (44.0)
Medical history by system category, *n* (%)^a^	
Cardiovascular disorders	44 (88.0)
Endocrine disorders	25 (50.0)
Gastrointestinal disorders	20 (40.0)
Genitourinary disorders	22 (44.0)
Hematopoietic/lymphocytic disorders	15 (30.0)
Musculoskeletal disorders	21 (42.0)
Respiratory disorders	19 (38.0)
Medical history by MedDRA preferred term, *n* (%)^a^	
Atrial fibrillation	12 (24.0)
Diabetes mellitus	10 (20.0)
Hypertension	36 (72.0)
Patients with underlying conditions, *n* (%)^b^	7 (18.9)
Malignancy	3 (42.9)
Autoimmune disease	1 (14.3)
Pregnancy/postpartum	1 (14.3)
Infection	1 (14.3)
Other	2 (28.6)

a ⩾10 patients in total.

b Percentages are based on the number of patients with available data (*n* = 37). Underlying conditions refer to those that are potentially associated with acquired hemophilia A.

MedDRA, Medical Dictionary for Regulatory Activities; rpFVIII, recombinant porcine factor VIII; SD, standard deviation.

At the start of rpFVIII treatment for the initial BE, almost all patients with available information had abnormal levels of hemoglobin [24/25 (96.0%)] and all patients had abnormal hematocrit [*n* = 21 (100.0%)] and red blood cell levels [*n* = 22 (100.0%)]. All patients reported at least one condition or event in their medical history. Seventeen patients (34%) had a history of malignancy; cases of malignancy were captured under different system categories, and the most frequently reported medical history by MedDRA terms are shown in Table 1. Before study entry, 60% (30/50) of patients had received immunosuppressive therapy to eradicate anti-FVIII inhibitors and/or hemostatic agents to treat bleeding (prior treatments are reported in Supplemental Table 1).

### Safety

Overall, 148 AEs were reported in 41 patients, of which 46 were SAEs (five SAEs were considered probably related to rpFVIII) (Table 2). In 11 patients, 12 AEs were reported as having a fatal outcome, none of which were assessed as being related to rpFVIII. Most AEs (*n* = 67) were considered as moderate in severity. There were no reports of hypersensitivity reactions, thromboembolic events, or dose dispensing medication errors.

**Table 2.** Overall incidence of AEs.

	Patients with ⩾1 event,^a^ *n*	Events, *n*	Incidence,^b^ estimate (95% CI) %
Any AE	41	148	82.00 (68.56–91.42)
SAEs^c^	30	46	60.00 (45.18–73.59)
Severity^d^			
Mild	20	38	40.00 (26.41–54.82)
Moderate	22	67	44.00 (29.99–58.75)
Anti-FVIII antibody increased	2	2	
Bladder tamponade	2	2	
Hypertension	2	3	
Pyrexia	2	2	
Renal impairment	2	2	
Urinary tract infection	2	2	
Severe	24	38	48.00 (33.66–62.58)
Anemia	2	2	
Anti-pFVIII inhibitors	2	2	
Death^e^	3	3	
Drug ineffective	2	2	
Hemorrhage	2	2	
Unknown	3	5	6.00 (1.25–16.55)
Relationship to rpFVIIId			
Not related	38	116	76.00 (61.83–86.94)
Unlikely related	3	19	6.00 (1.25–16.55)
Possibly related^f^	1	1	2.00 (0.05–10.65)
Probably related^g^	8	9	16.00 (7.17–29.11)
Missing	1	3	2.00 (0.05–10.65)

a Preferred terms are shown for moderate and severe AEs in ⩾2 patients.

b Incidence proportion is defined as number of patients with at least one event in a category/total number of patients.

c Five SAEs in five patients were considered probably related to rpFVIII: lack of rpFVIII efficacy, drug resistance due to FVIII inhibition, antibody test positive for anti-pFVIII inhibitors (one event per patient), and *de novo* anti-pFVIII inhibitors in two patients.

d If a patient experienced multiple events that mapped to the same MedDRA preferred term, the most severe or the most related occurrence was counted in the summary.

e Three deaths were considered as AEs in the study; two listed as ‘death’ and one as ‘sudden cardiac death’.

f Possibly related AEs: one moderate severity.

g Probably related AEs: three mild, one moderate, four severe, one unknown severity.

AE, adverse event; CI, confidence interval; FVIII, factor VIII; MedDRA, Medical Dictionary for Regulatory Activities; rpFVIII, recombinant porcine factor VIII; SAE, serious adverse event.

Among the 145 AEs with information on their relationship to rpFVIII, 9 were considered probably related to rpFVIII, 1 AE was considered possibly related, and 135 AEs were unrelated/unlikely related. Five patients had probably related AEs that were suggestive of a lack of efficacy/effect according to the standardized MedDRA query (SMQ) for lack of efficacy (SMQs are validated, standard sets of MedDRA terms,^15^ Table 3). In these patients, the initial reported rpFVIII dose was lower than the recommended dose of 200 U/kg^9,10^ (between 54 and 180 U/kg). Four patients had probably related AEs that were classified as immunogenicity AESIs. These were drug resistance due to FVIII inhibition in one patient; antibody test positive for anti-pFVIII inhibitors in one patient (no increase in anti-pFVIII inhibitor titer from a measurable baseline); and *de novo* anti-pFVIII inhibitors in two patients. The possibly related AE was an increase in anti-hFVIII inhibitor (Table 3).

**Table 3.** AEs that were possibly or probably related to rpFVIII.

	Patients, *n* (incidence proportion)	Events
Probably related AEs		
Drug ineffective^a^	3 (6.0)	3
Therapy nonresponder^a^	1 (2.0)	1
Drug resistance due to FVIII inhibition^a,b^	1 (2.0)	1
Antibody test positive for anti-pFVIII inhibitors^b,c^	1 (2.0)	1
*De novo* anti-pFVIII inhibitors^b^	2 (4.0)	2
Hypertensive crisis^d^	1 (2.0)	1
Possibly related AEs		
Increase in anti-hFVIII inhibitor	1 (2.0)	1

a Included as an SMQ for lack of efficacy (SMQs are validated, standard sets of MedDRA terms^15^).

b Classified as an immunogenicity AESI.

c Defined as an increase in anti-pFVIII inhibitor titer from a measurable baseline.

d Resolved and was later assessed as not related to rpFVIII.

AE, adverse event; AESI, AEs of special interest; MedDRA, Medical Dictionary for Regulatory Activities; rpFVIII, recombinant porcine factor VIII; SMQ, standardized MedDRA query.

### Secondary objectives

#### Anti-FVIII inhibitors

Testing for anti-pFVIII inhibitors was performed in a small number of patients with initial BEs. Anti-pFVIII titers remained stable during and after rpFVIII treatment in comparison with baseline titers (Table 4). Using a quantification limit of 0.6 BU/mL as specified in the World Federation of Hemophilia guidelines,^16^ 11 patients had detectable anti-rpFVIII titers during or after rpFVIII treatment (Table 4), with a median [range, interquartile range (IQR)] treatment duration of 3.04 (0.4–60.9, 1.24–8.69) days. Anti-pFVIII immunogenicity data at baseline and after rpFVIII treatment were analyzed in relation to BE outcomes, but only 6 patients had available data for change from baseline. No specific trend or correlation between immunogenicity and bleed outcomes could be established due to the small number of patients for whom these data were available. Physicians were also asked about immunogenicity in the electronic case report form (eCRF) with the question: ‘Did the patient develop an inhibitor against pFVIII or increased inhibitor level from pretreatment values during or following OBIZUR treatment?’ Twenty-six responses were received [immunogenicity, *n* = 4 (15.4%); no immunogenicity, *n* = 22 (84.6%)], and data were missing for 24 patients.

**Table 4.** Inhibitor assessments for initial BEs treated with rpFVIII.

	Before rpFVIII^a^	During rpFVIII^b,c^	After rpFVIII^b,c^
Anti-pFVIII inhibitor titer for all available measurements, BU/mL			
*n*	10	11	9
Median (IQR)	1.0 (0.2–8.0)	1.1 (0.3–26.1)	0.9 (0.0–2.5)
Anti-pFVIII inhibitor titer (⩾0.6 BU/mL),^d^ BU/mL			
*n*	4	7	5
Median (IQR)	4.6 (1.2–79.0)	3.7 (1.1–35.0)	2.5 (1.5–4.5)
Range	1.1–150	0.8–179	0.9–39
Anti-hFVIII inhibitor titer, BU/mL			
*n*	38	25	39
Median (IQR)	9.3 (3.6–50.0)	8.0 (3.2–18.2)	3.9 (1.0–11.8)
Range^e^	0.4–999.99	0.4–449.32	0–999.99

Values >1000 BU/mL were recorded as ‘999.99 BU/mL’ due to the digit limit in the relevant eCRF field.

a The assessment closest to and before the first rpFVIII infusion was used for the initial BE.

b The assessment with the highest value for this period was used.

c ‘During rpFVIII’ represents the time period between the first and last rpFVIII infusion for the initial BE. ‘After rpFVIII’ represents the time period after the last rpFVIII infusion for the initial BE and until the subsequent bleeding event treated with rpFVIII.

d Only one patient had data for both during and after rpFVIII periods.

BE, bleeding event; BU, Bethesda units; eCRF, electronic case report form; hFVIII, human factor VIII; IQR, interquartile range; *n*, number of patients with available data; pFVIII, porcine factor VIII; Q1, 25th percentile; Q3, 75th percentile; rpFVIII, recombinant porcine factor VIII.

Overall, lower median anti-hFVIII inhibitor titers for initial BEs were reported after rpFVIII treatment than at baseline (Table 4). Of 32 patients with anti-hFVIII inhibitor measurements available at baseline and after treatment, 10 had an increase in anti-hFVIII inhibitor titer after rpFVIII administration. However, the IQR indicated that the majority of anti-hFVIII titers were lower after rpFVIII, with an IQR of 1.0–11.8 BU/mL compared with 3.6–50.0 BU/mL at baseline.

#### FVIII assessments

For initial BEs, few patients had FVIII:C measured within 2 h and between 2 and 4 h after first rpFVIII administration (*n* = 22 and *n* = 4, respectively) (Table 5). The median FVIII:C at the measurement closest to rpFVIII administration for the initial BE (up to 2 h before administration) was 2.5% (*n* = 8), which gradually increased from the start of treatment through 4 h after rpFVIII administration, with a median FVIII:C of 132.0% (*n* = 49) beyond 4 h.

**Table 5.** FVIII:C after rpFVIII treatment for initial BEs.

FVIII:C, %	Up to 2 h before first rpFVIII infusion^a^	Within 2 h after first rpFVIII infusion^b^	Within 2–4 h after first rpFVIII infusion^b^	Beyond 4 h of first rpFVIII infusion^b^
*n*	8	22	4	49
Median	2.5	58.0	54.5	132.0
IQR	1.0–47.0	18.0–95.0	47.5–146.5	68.4–225.3
Range	1–94	0–161	44–235	0.1–407
Missing^c^	2	0	0	1
Not assessed^c^	40	28	46	0

a The assessment closest to and before the first rpFVIII infusion was used for the initial BE.

b The assessment with the highest value for this period was used.

c The number of patients with missing data or without an assessment within the defined time period.

BE, bleeding event; FVIII, factor VIII; FVIII:C, factor VIII activity; IQR, interquartile range; *n*, number of patients with available data; rpFVIII, recombinant porcine factor VIII.

#### Hemostatic effectiveness

##### Bleeding characteristics

**Figure 2.**
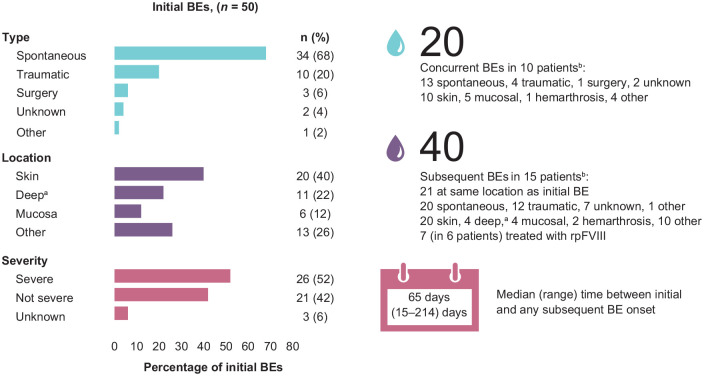
Bleeding characteristics. BE, bleeding event; rpFVIII, recombinant porcine factor VIII. ^a^Musculoskeletal or retroperitoneal areas. ^b^New BEs were classified as concurrent BEs if they occurred at a different anatomical location during treatment or within 72 h of the resolution of the previous BE, or as subsequent BEs if they occurred >72 h after the resolution of the previous BE, regardless of the anatomical location.

Most initial BEs and half of subsequent BEs were spontaneous. Rebleeds occurred with two initial and two subsequent BEs (one rebleed in each patient; Figure 2).

##### Bleed resolution with rpFVIII

**Figure 3.**
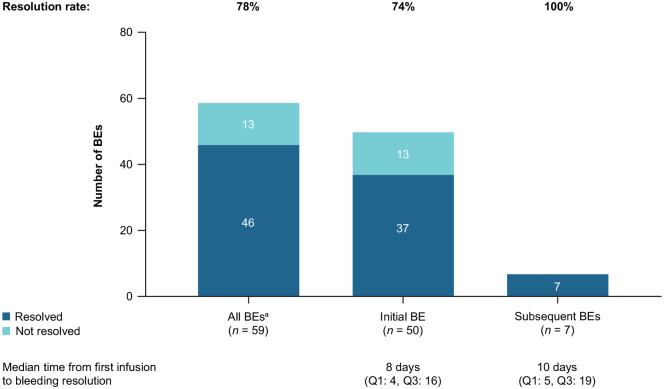
Bleed resolution with rpFVIII. ^a^All bleeds = 50 initial BEs + 7 subsequent BEs + 1 rebleeding + 1 concurrent BE. BE, bleeding event; Q1, 25th percentile; Q3, 75th percentile; rpFVIII, recombinant porcine factor VIII.

More than half of patients received rpFVIII as first-line treatment of initial BEs. Treatment details for initial and subsequent BEs are available in Table 6. Of the 50 initial BEs, 37 (74.0%) resolved after the rpFVIII treatment course [median (IQR) time from first infusion to bleeding resolution: 8.0 (4.0–16.0) days; Figure 3]. Among the 37 resolved initial BEs, 35 resolved after an initial rpFVIII treatment course and 2 after a subsequent rpFVIII treatment course. Most resolved initial BEs were spontaneous (70%, *n* = 26) followed by traumatic (16%, *n* = 6), surgical (5%, *n* = 2), unknown/other (8%, *n* = 3). were severe, 51% (*n* = 19). Of the resolved initial BEs, 43% (n=16) were not severe, and 2 were of unknown severity. Of the seven subsequent BEs reported in six patients, all BEs resolved after rpFVIII treatment.

**Table 6.** rpFVIII treatment details for initial and subsequent BEs.

	Initial BEs (*n* = 50)	Subsequent BEs (*n* = 7)
rpFVIII used as first-line therapy, *n* (%)	29 (58.0)	–
Time from initial/subsequent BE to first infusion,^a^ days	8.0 (1–83)	1.0 (1–3)
IQR	2.0–22.0	1.0–3.0
Treatment duration,^b^ h	79.0 (0–1703)	144.0 (0–459)
IQR	36.0–243.4	39.0–430.5
Reported first dose,^c^ U/kg	54.0 (11–200)	63.0 (31–80)
IQR	45.0–100.0	46.0–77.0
Reported total dose,^d^ U/kg	400.0 (45–8403)	641.0 (80–1980)
IQR	208.0–764.0	197.0–1051.0
Average reported dose over consecutive infusions,^e^ U/kg	54.0 (12–180)	63.0 (31–80)
IQR	40.9–73.5	39.4–70.7
Number of infusions^f^	8.0 (1–136)	13.0 (1–28)
IQR	3.0–17.0	3.0–25.0

Data are median (range) unless otherwise stated.

a First infusion date/time, minus initial bleeding date/time, plus 1.

b Date/time of last infusion minus date/time of first infusion.

c The median first dose of rpFVIII in this study was lower than that recommended in the current EMA and US FDA labels (200 U/kg). The EMA label also states that if testing of anti-pFVIII antibodies is negative at baseline, a rpFVIII dose lower than 200 U/kg may be used as the initial treatment dose.

d The sum of reported doses across infusions per patient for the initial BE.

e Total reported dose divided by number of infusions.

f Count of first infusion plus subsequent infusions for the initial BE.

BE, bleeding event; EMA, European Medicines Agency; FDA, Food and Drug Administration; IQR, interquartile range; *n*, number of patients with observations; rpFVIII, recombinant porcine factor VIII.

Some trends in the descriptive data were observed between the resolved and unresolved groups. Initial BEs resolved in a higher proportion of patients who received rpFVIII as first-line therapy (83%, *n* = 24/29) than in patients who received it as second-line therapy (62%, *n* = 13/21). The median (IQR) time from initial BE to first rpFVIII infusion was shorter for patients with resolved BEs *versus* unresolved BEs [6.5 (2.0–15.5) *versus* 24.0 (4.0–28.0) days, respectively]. The median (IQR) reported first dose of rpFVIII for patients with resolved BEs was 74.0 (45.0–100.0) U/kg compared with 50.0 (49.0–80.0) U/kg for unresolved BEs.

#### Concomitant medication

Concomitant treatment during the study was reported by all enrolled patients (Supplemental Table 1).

#### Inhibitor eradication

Data from patients with inhibitor eradication based on the physician’s judgment (none, partial, or complete remission) were available during or after rpFVIII treatment for initial and subsequent BEs. Of 46 initial BEs with available data, complete remission was achieved for 14 (30.4%) BEs, partial remission for 13 (28.3%) BEs, and no remission for 19 (41.3%) BEs. Six of the seven subsequent BEs treated with rpFVIII had information on inhibitor eradication. Three (50.0%) of these BEs achieved complete remission, one (16.7%) partial remission, and the remaining two (33.3%) no remission. Considering the response for each patient after the initial BE (as each patient had one initial BE), 58.7% of patients achieved partial or complete inhibitor eradication after their initial BE.

### Exploratory objective

The proportion of patients with inhibitors was numerically higher among patients whose initial bleeding did not resolve, compared with patients whose bleeding resolved, but patient numbers were small and the difference was not significant [2/10 (20.0%) and 2/16 (12.5%), respectively, *p* = 0.6254].

## Discussion

The results of this PASS are informative of real-world treatment of AHA with rpFVIII. No new safety signals were observed when compared with the phase II/III clinical trial of rpFVIII in AHA (NCT01178294).^14^ There were no reported AEs of hypersensitivity reactions or thromboembolic events, which are potential safety concerns with rpFVIII, and no dose dispensing medication errors were reported. These findings are consistent with several case-series studies reporting treatment of AHA with rpFVIII in real-world settings, in which no related AEs were reported.^17–21^

Of the 50 initial BEs treated with rpFVIII, 74.0% resolved, even when first doses were lower than recommended in the European Medicines Agency (EMA) and US Food and Drug Administration labels (200 U/kg).^9,10^ The median reported first dose was 54 U/kg, with 75% of patients receiving doses <100 U/kg. The administration of lower than recommended first doses has also been reported in other publications summarizing real-world experience in different centers.^17,20–22^ Of note, the EMA label has been updated to state that if anti-rpFVIII antibody testing is negative at baseline, a dose lower than the recommended 200 U/kg can be used as the initial treatment dose.^10^

Some trends were noted regarding BE resolution, although no statistical testing was performed on these data. The data suggest that a shorter time from the initial bleed to the first rpFVIII infusion could favor bleed resolution. In addition, a higher proportion of patients receiving first-line rpFVIII achieved resolution of their initial BE than those receiving second-line therapy, suggesting that first-line rpFVIII is potentially better than second-line therapy for resolving initial BEs. Alternatively, this may reflect that BEs in patients requiring second-line therapy are more treatment-refractory than those requiring first-line therapy. Similarly, in the phase II/III clinical trial (NCT01178294),^14^ patients treated with rpFVIII as first-line therapy had a higher rate of treatment success than patients treated with another hemostatic agent before rpFVIII treatment.

Based on a case-series study of four patients with AHA and one with congenital hemophilia with inhibitors, conducted by Abou-Ismail *et al*.,^17^ it was suggested that anti-pFVIII inhibitors may lead to resistance to rpFVIII after exposure to rpFVIII. In the current study, the association between BE resolution and the development of *de novo* anti-pFVIII inhibitors or increased anti-pFVIII inhibitor titer from baseline was not statistically significant. However, this analysis was limited by the infrequent assessment of anti-pFVIII inhibitors, which precludes a conclusion from this association. This observation also suggests that physicians at the study sites do not routinely monitor anti-pFVIII inhibitor titer in the treatment of patients with AHA. Only two of the five AEs that were part of the SMQ for lack of efficacy (drug resistance and therapy nonresponder) occurred in patients who also had immunogenicity AESIs. In the five patients with AEs that were part of the lack of efficacy SMQ, the first rpFVIII dose was lower than the recommended dose of 200 U/kg (40–180 U/kg).

FVIII:C was monitored for a limited number of patients before the first rpFVIII infusion and within the first 4 h after the infusion, despite the recommendation in the summary of product characteristics to monitor FVIII:C at 30 min after the first infusion and at 3 h after completing rpFVIII therapy.^10^ This might reflect challenges encountered in real-world hospital settings. Time to initial bleeding resolution (8.0 days) was similar to the 6.5 days in the phase II/III clinical trial (NCT01178294).^14^ However, in this PASS, BE control was assessed on the basis of bleeding resolution according to physician judgment, whereas in the clinical trial, it was measured using body system-specific evaluation criteria.^14^

The mean age of the enrolled patients (76 years) is consistent with AHA mainly affecting older people,^2^ and suggests that the data may be generalizable to the wider AHA population. The 60:40 ratio of males to females in the patient population is consistent with other AHA studies from Europe and Taiwan.^23–25^ There were several limitations, as expected given the study’s observational nature. These included the fact that not all assessments of interest were routinely collected or collected in a standardized manner. The availability of FVIII:C data and anti-pFVIII inhibitor assessments were limited, and *in vivo* post-infusion FVIII recovery and trough levels were not captured. This impacts the ability to fully assess the link between dosing and immunogenicity. In addition, AHA can be a secondary complication of checkpoint inhibitors, but these were also not captured. Researchers have questioned whether rpFVIII increases anti-hFVIII titers in some patients. The case-series study by Abou-Ismail *et al.*^17^ found that three of four patients with AHA had an increase of up to four-fold in anti-hFVIII inhibitor titers after receiving rpFVIII. The data available from the current study to answer this question were limited. Overall, lower median anti-hFVIII titers were reported after than before initial rpFVIII treatment.

An additional study limitation was that the assessment of effectiveness and inhibitor eradication was based on the investigator’s judgment, and the analysis of inhibitor eradication data was linked to BEs rather than individual patients, thus limiting the analysis of follow-up to remission at the patient level. As race or ethnicity was not available for 22 of the 50 patients enrolled (44%) it is difficult to make any conclusions regarding applicability to other populations. Finally, this study did not capture clinician and patient satisfaction with the ability of rpFVIII to control bleeding quickly. Metrics are needed that capture the speed of BE control as well as satisfaction with delivery and receipt of a treatment that quickly stabilizes serious patient bleeding.

## Conclusion

No new safety signals were observed in this real-world study. The findings of this study support the use of rpFVIII as first-line treatment for AHA and align with findings from the phase II/III clinical trial (NCT01178294) of rpFVIII in the treatment of BEs in adults with AHA.^14^

## Supplemental Material

sj-docx-1-tah-10.1177_20406207241260332 – Supplemental material for Real-world use of recombinant porcine sequence factor VIII in the treatment of acquired hemophilia A: EU PASS Supplemental material, sj-docx-1-tah-10.1177_20406207241260332 for Real-world use of recombinant porcine sequence factor VIII in the treatment of acquired hemophilia A: EU PASS by Wolfgang Miesbach, Nicola Curry, Paul Knöbl, Charles Percy, Rita Santoro, Alvin H. Schmaier, Karolin Trautmann-Grill, Kayode Badejo, Jie Chen, Masoud Nouri, Pooja Oberai and Robert Klamroth in Therapeutic Advances in Hematology

## Declarations

**Ethics approval and consent to participate:** The study protocol and informed consent form were reviewed and approved by institutional review boards or independent ethics committees and applicable regulatory authorities at each study site.

**Consent for publication:** Not applicable.

**Availability of data and materials:** The datasets, including the redacted study protocol, redacted statistical analysis plan, and individual participant data supporting the results reported in this article, will be made available within 3 months from the initial request to researchers who provide a methodologically sound proposal. The data will be provided after its deidentification, in compliance with applicable privacy laws, data protection, and requirements for consent and anonymization. The study sponsor analyzed the data and conducted the statistical analyses.
