# Corrigendum to “Real-world use of recombinant porcine sequence factor VIII in the treatment of acquired hemophilia A: EU PASS”

**DOI:** 10.1177/20406207241288442

**Published:** 2024-10-04

Miesbach W, Curry N, Knöbl P, et al. Real-world use of recombinant porcine sequence factor VIII in the treatment of acquired hemophilia A: EU PASS. *Therapeutic Advances in Hematology*. 2024;15. doi:10.1177/20406207241260332

In the above-mentioned article, the text “were severe, 51% (*n* = 19). Of the resolved initial BEs, 43% (n=16) were not severe, and 2 were of unknown severity.” has been corrected to “Of the resolved initial BEs, 43% (n=16) were severe, 51% (*n* = 19) were not severe, and 2 were of unknown severity.” under the heading “*Bleed resolution with rpFVIII*” of the “Result” section.

The article has been updated to reflect the corrections.

