# Supplementary material for: Real-world use of recombinant porcine sequence factor VIII in the treatment of acquired hemophilia A: EU PASS
Source: Ther Adv Hematol. 2024 Sep 2;15:20406207241260332. doi: 10.1177/20406207241260332 (PMC11369870; doi:10.1177/20406207241260332)
Supplement: sj-docx-1-tah-10.1177_20406207241260332 – Supplemental material for Real-world use of recombinant porcine sequence factor VIII in the treatment of acquired hemophilia A: EU PASS [file sj-docx-1-tah-10.1177_20406207241260332.docx]

**Real-world use of recombinant porcine sequence factor VIII in the treatment of acquired hemophilia A: EU PASS**

Wolfgang Miesbach, Nicola Curry, Paul Knöbl, Charles Percy, Rita Santoro, Alvin H. Schmaier, Karolin Trautmann-Grill, Kayode Badejo, Jie Chen, Masoud Nouri, Pooja Oberai and Robert Klamroth

**SUPPORTING INFORMATION**

# Supplementary Figure 1. Study design.


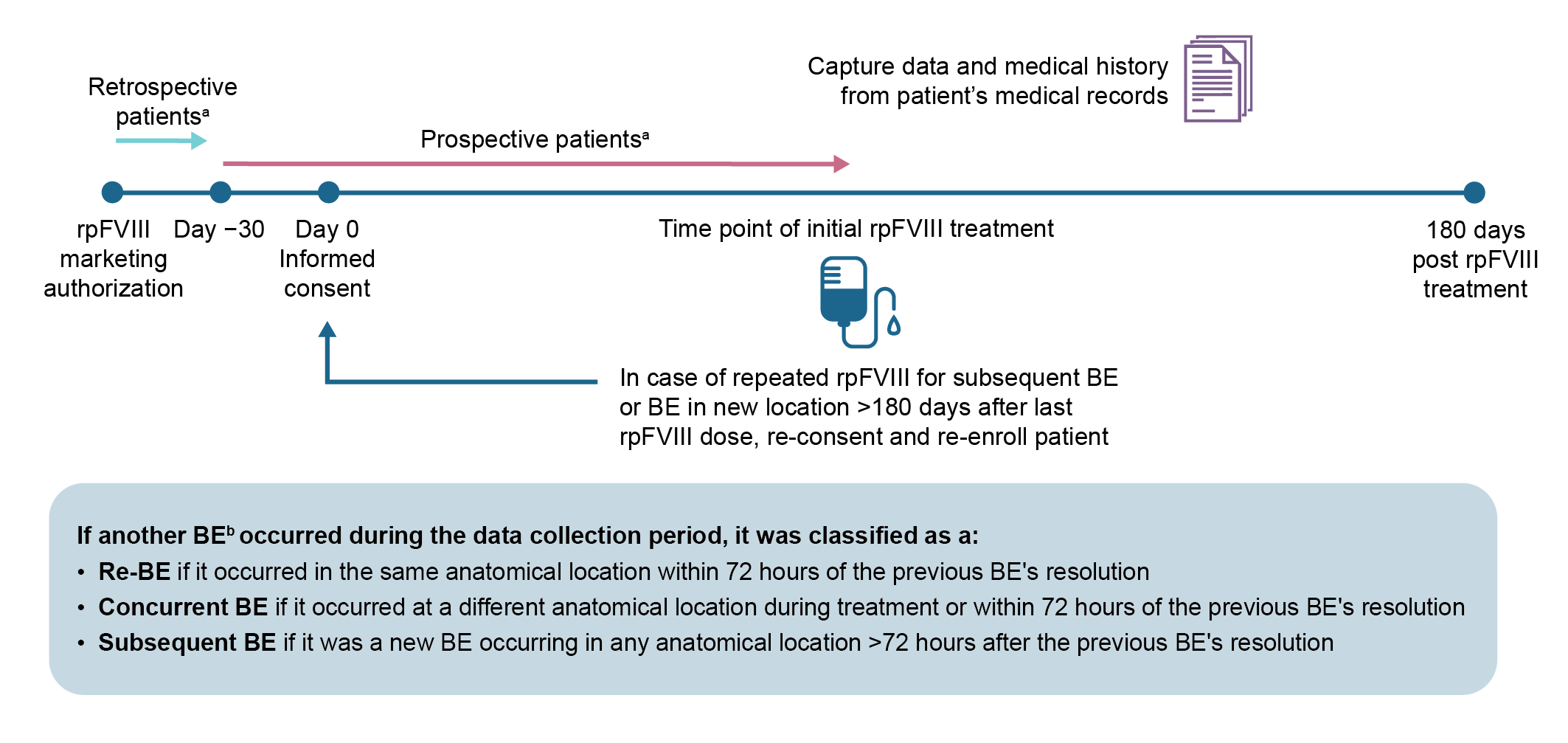


BE, bleeding event; rpFVIII, recombinant porcine factor VIII.

^a^A retrospective patient was treated with rpFVIII between its marketing authorization and >30 days before the date of the informed consent. All patients who did not have to give informed consent owing to local regulations or who did not have a screening visit (e.g. deceased patients) were also considered as retrospective patients. A prospective patient was being treated or had been treated after providing informed consent within 30 days of starting rpFVIII administration.

^b^The patient was re-consented and re-enrolled in the study using the same subject identifier in the event of repeated treatment with rpFVIII for a subsequent bleed or a new bleed in a new location if >180 days after the last rpFVIII treatment.

# Supplementary Table 1. Prior and concomitant treatments.

|  | ***N* = 50**  ***n* (%)** |
| --- | --- |
| Prior treatment^a^ | 30 (60.0) |
| To treat a bleed event | 26 (86.7)^b^ |
| Concentrated red blood cells | 12 (24.0) |
| Factor VIII inhibitor bypassing fraction | 9 (18.0) |
| Eptacog alfa (activated) | 8 (16.0) |
| Tranexamic acid | 3 (6.0) |
|  |  |
| Plasma | 3 (6.0) |
| Prothrombin complex concentrate | 2 (4.0) |
| Concentrated platelets | 2 (4.0) |
| To eradicate anti-FVIII inhibitors | 12 (40.0)^b^ |
| Prednisolone | 8 (16.0) |
| Rituximab | 2 (4.0) |
| Methylprednisolone | 2 (4.0) |
| Concomitant medication^c^ | 50 (100) |
| To treat AE/SAEs | 23 (46.0) |
| To treat a bleeding event | 27 (54.0) |
| Activated eptacog alfa | 11 (22.0) |
| FVIII inhibitor bypassing fraction | 9 (18.0) |
| Tranexamic acid | 7 (14.0) |
| Prophylaxis of bleeding | 13 (26.0) |
| Tranexamic acid | 7 (14.0) |
| To eradicate anti-FVIII inhibitors^d^ | 38 (76.0) |
| Prednisolone | 25 (50.0) |
| Cyclophosphamide | 13 (26.0) |
| Rituximab | 11 (22.0) |
| Methylprednisolone | 5 (10.0) |
| Prednisone | 5 (10.0) |

AE, adverse event; eCRF, electronic case report form; FVIII, factor VIII; rpFVIII, recombinant porcine factor VIII; SAE, serious adverse event.

*n* indicates number of patients with observations; *N* indicates number of enrolled patients.

^a^Prior treatment refers to a positive response to the eCFR question “has the patient previously received any immunosuppressive therapy to eradicate anti-FVIII inhibitors and/or haemostatic agents to treat bleeding before taking rpFVIII?” Treatments are not mutually exclusive and are specified if used by ≥4% of patients.

^b^Percentage among 30 patients who previously received any immunosuppressive and/or haemostatic agents.

^c^Concomitant medications include those that were ongoing or given after the initial rpFVIII administration and are specified if used by ≥10% of patients.

^d^Overall, glucocorticoids were used concomitantly by 64% (*n* = 32/50) of patients to eradicate anti-FVIII inhibitors.
